# A Systems Biology Approach to Identifying a Master Regulator That Can Transform the Fast Growing Cellular State to a Slowly Growing One in Early Colorectal Cancer Development Model

**DOI:** 10.3389/fgene.2020.570546

**Published:** 2020-10-08

**Authors:** Jihye Choi, Jeong-Ryeol Gong, Chae Young Hwang, Chang Young Joung, Soobeom Lee, Kwang-Hyun Cho

**Affiliations:** Laboratory for Systems Biology and Bio-inspired Engineering, Department of Bio and Brain Engineering, Korea Advanced Institute of Science and Technology (KAIST), Daejeon, South Korea

**Keywords:** colorectal cancer, adenomatous polyposis coli, single cell transcriptomics, gene regulatory network, CCDC85B, systems biology, master regulator analysis

## Abstract

Colorectal cancer (CRC) has been most extensively studied for characterizing genetic mutations along its development. However, we still have a poor understanding of CRC initiation due to limited measures of its observation and analysis. If we can unveil CRC initiation events, we might identify novel prognostic markers and therapeutic targets for early cancer detection and prevention. To tackle this problem, we establish the early CRC development model and perform transcriptome analysis of its single cell RNA-sequencing data. Interestingly, we find two subtypes, fast growing vs. slowly growing populations of distinct growth rate and gene signatures, and identify CCDC85B as a master regulator that can transform the cellular state of fast growing subtype cells into that of slowly growing subtype cells. We further validate this by *in vitro* experiments and suggest CCDC85B as a novel potential therapeutic target that may prevent malignant CRC development by suppressing stemness and uncontrolled cell proliferation.

## Introduction

Colorectal cancer (CRC) has been most extensively studied for characterizing genetic mutations along its development. Loss of adenomatous polyposis coli (APC) is considered as the first step of CRC development, which is followed by mutations of other driver genes such as KRAS and TP53 (Fearon and Vogelstein, 1990; Powell et al., 1992). Gene alterations of APC abrogate its binding with β-catenin and result in β-catenin release, which in turn brings about hyper-activity of the canonical Wnt signaling pathway and failure of the cell-cell adhesion regulation (Valenta et al., 2012). The disruption of APC ultimately leads to dysfunction in maintaining the homeostasis of cellular regulation and results in more chances of other genetic alterations. Our understanding of CRC progression has been advanced over last few decades, but we still do not know much about its initiation process starting from APC deficiency. This is because there are limited measures for observation and analysis of cancer initiation events. If we unveil CRC initiation events, we might be able to identify novel prognostic markers and therapeutic targets for early cancer detection and prevention (Kaufman et al., 2016).

In order to investigate the cancer initiation process, we need to utilize a tool that can monitor instantaneous and delicate changes of the transcriptomic landscape during the initiating events. Single cell RNA sequencing (scRNA-seq) can fulfill this demand by dissecting gene expressions at each individual cellular resolution. Several studies on development and oncology exemplified that we can capture heterogeneity in cell fate decision or drug response using scRNA-seq (Maamar et al., 2007; Huang, 2009; Eldar and Elowitz, 2010; Petropoulos et al., 2016).

As it is not possible to analyze or understand all uncountable miniscule changes at an mRNA level captured by scRNA-seq, it is essential to find out a few key genes that might be primarily responsible for controlling the cell phenotypes. Such genes, called “master regulators,” trigger a series of gene regulation events which ultimately lead to critical changes in gene regulatory networks (Califano and Alvarez, 2017). We note that recent progresses in systems biology show the importance of unraveling the gene regulatory network and the causal relationships among the gene regulations to properly understand the complex biological phenomena (Schmidt et al., 2005; Kim and Cho, 2006; Park et al., 2006; Kim et al., 2007, 2011; Kwon and Cho, 2007; Murray et al., 2010). Previous studies report that master regulator analysis can successfully identify crucial genes for maintaining and controlling cancer gene regulatory networks (Wang et al., 2009; Campbell et al., 2016).

In this study, to understand the earliest events in CRC initiation, we establish an early CRC development model by disrupting APC in the normal human colorectal epithelial cell with shRNA and conduct scRNA-seq. Interestingly, we find two subtypes, fast growing vs. slowly growing populations of distinct growth rate and gene signatures. We focus on how they work differently at the transcriptomic level and conduct master regulator analysis. As a result, we find CCDC85B as a master regulator that can transform the cellular state of fast growing subtype cells into that of slowly growing subtype cells. We further validate this by *in vitro* experiments and suggest a novel therapeutic strategy that may prevent malignant CRC development by suppressing stemness and uncontrolled cell proliferation.

## Materials and Methods

### Cell Culture

Immortalized human colon epithelial cells (HCEC), 1CT and its wild type APC depleted version, 1CT-A cells are generously provided by Jerry W. Shay (University of Texas, Dallas, TX, United States). 1CT and 1CT-A cells are cultured in basal X media (DMEM: M199, 4:1; WelGENE Inc., Gyeongsan, Korea), supplemented with epidermal growth factor (20 ng·ml^−1^; Thermo Fisher Scientific, Waltham, MA, United States), hydrocortisone (1 mg·ml^−1^), insulin (10 mg·ml^−1^), transferrin (2 mg·ml^−1^), sodium selenite (5 nM; all from Sigma, Deisenhofen, Germany), 2% FBS, and antibiotics (100 units·ml^−1^ of penicillin, 100 μg·ml^−1^ streptomycin, and 0.25 μg·ml^−1^ of Fungizone; Life Technologies Corp., Carlsbad, CA, United States). Cells are cultured at 37°C in a humidified atmosphere containing 5% CO_2_.

### Transfection and Transduction of shRNA

For lentivirus production, HEK 293T cells are transfected with shRNA targeting APC (shAPC; TRCN0000244294, Sigma) and packaging mix (pLP1, pLP2, and pLP/VSVG) using Lipofectamine (Invitrogen, Waltham, MA, United States), according to manufacturer’s protocols. Then viral supernatants are collected and applied to target cells with polybrene (4 μg·ml^−1^; Sigma). Infected cells are selected with puromycin (500 ng·ml^−1^; Sigma) before harvest. 1CT cells infected with scrambled shRNA (shScr) are prepared as control samples, and their culture periods are matched with the shAPC samples.

### Transfection of siRNA

Control siRNA (siControl), CCDC85B siRNA (siCCDC85B-1 and siCCDC85B-2), and PTTG1 siRNA (siPTTG1-1 and siPTTG1-2) oligonucleotides (BIONEER Corporation, Daejeon, South Korea) are synthesized in a sense-antisense duplex form (Table 1). Primer sequences for ASCL2, CCDC85B, CCNE1, and CCNA2 were referred from OriGene Technologies, Inc. (Rockville, MD, United States). For siRNA transfection, mixture of siRNAs and RNAiMAX (Thermo Fisher Scientific) with final concentration of 2 μM is applied to the target cell on the 60 mm culture dish, following the manufacturer’s protocol. After 24 h, transfected cells are subcultured into 24-well plates and 60 mm culture dish for growth curve check and RNA harvest, respectively.

**Table 1:** Sequences of siRNAs.

siRNA name	Target gene	Sense siRNA sequence (5'-3')	Antisense siRNA sequence (5'-3')
siCCDC85B-1	CCDC85B	GAGGUUCGAAGCUCCUAGU	ACUAGGAGCUUCGAACCUC
siCCDC85B-2	CCDC85B	GAUUGGCUGUCCUUCCAUA	UAUGGAAGGACAGCCAAUC
siPTTG1-1	PTTG1	AGCACCAGAUUGCGCACCU	AGGUGCGCAAUCUGGUGCU
siPTTG1-2	PTTG1	GUUGAAUUGCCACCUGUUU	AAACAGGUGGCAAUUCAAC

### Total RNA Extraction and qRT-PCR

Total RNA is extracted from cells by using RNA-spin™ Total RNA Extraction Kit (iNtRON Biotechnology, Gyeonggi, South Korea), according to the manufacturer’s protocol, and treated with RNase-free DNase I (Thermo Fisher Scientific) to remove contaminating genomic DNA. cDNA is then synthesized from total RNA by reverse transcription (RT) using a DiaStar RT kit (Solgent, Daejeon, Korea) and the PCR system (Veriti 96-well Thermal Cycler; Applied Biosystems, Waltham, MA, United States). Quantitative reverse transcription PCR (qRT-PCR) analysis is performed using the QuantStudio 5 real-time PCR system (Applied Biosystems) with the corresponding primers (Table 2).

**Table 2:** Sequences of qRT-PCR primers.

Target gene	Forward primer sequence (5'-3')	Reverse primer sequence (5'-3')
β actin	AGAGCTACGAGCTGCCTGAG	AGCACTGTGTTGGCGTACAG
APC	GCCCACGAATTCTAAAACCA	TTGTCCTGCCTCGAGAGATT
MYC	GTCAAGAGGCGAACACAC	TTGGACGGACAGGATGTA
CCDC85B	TCATGCAGGAGGTGAATCGGCA	AGTCCAGGAAGCAGCAGAGGTC
PTTG1	GGACCCCTCAAACAAAAACA	GAGAGGCACTCCACTCAAGG
CCNA2	CTCTACACAGTCACGGGACAAAG	CTGTGGTGCTTTGAGGTAGGTC
CCNB1	TTGGTGTCACTGCCATGTTT	CCGACCCAGACCAAAGTTTA
CCND1	GCTGCGAAGTGGAAACCATC	CCTCCTTCTGCACACATTTGA
CCNE1	TGTGTCCTGGATGTTGACTGCC	CTCTATGTCGCACCACTGATACC
LGR5	CTCCCAGGTCTGGTGTGT	GAGGTCTAGGTAGGAGGTGAAG
ASCL2	CGCCTACTCGTCGGACGA	GCCGCTCGCTCGGCTTCCG

### Bulk RNA Sequencing

RNA sequencing experiments are performed using tools from the commercial microarray service Ebiogen, Inc. (Seoul, Korea). Total RNA is extracted from 1CT and 1CT-A cells using RNA-spin™ (iNtRON) according to the manufacturer’s instructions. The isolated RNA is amplified and subjected to cDNA microarray (Ebiogen).

### Single Cell RNA Sequencing

HCEC-1CT cells infected with shAPC and their matched cells infected with shScr are harvested at 3- and 7-days after transduction, and stored on ice in PBS before the single cell library preparation. scRNA-seq is performed using the 10x Genomics Chromium V3 kit, following the manufacturer’s protocol (Zheng et al., 2017). We align the scRNA-seq dataset along hg38 with CellRanger 3.0.0, and process with Seurat 3.1 R toolkit (Butler et al., 2018; Stuart et al., 2019). We perform initial quality control with Seurat, following the standard preprocessing workflow for scRNA-seq data (Ilicic et al., 2016). Dying cells and multiplets are excluded under the assumption that unhealthy cells tend to have either very few genes (<200) or low unique feature counts (<500; Supplementary Figure S1 and Supplementary Table S1).

For batch correction, we use ComBat from surrogate variable analysis (sva) package (Johnson et al., 2007; Leek et al., 2012) on Trimmed Mean of M-values (TMM) normalized data. In addition, we check the consistency of the batch correction results by comparing the results of ComBat and the different method called canonical correlation analysis (CCA; Supplementary Figure S10).

Then we additionally conduct data imputation using deep count autoencoder (DCA) in order to denoise scRNA-seq datasets (Eraslan et al., 2019), and compare the scRNA-seq datasets before and after denoising process with DCA (Supplementary Figure S8). By using another data imputation tool, Adaptively-thresholded Low Rank Approximation (ALRA), we check the consistency of the imputation performance (Linderman et al., 2018; Supplementary Figure S9).

Single cell gene expression levels are scaled, so that the mean is equal to zero and the variance is equal to one, and the effects of cell cycle heterogeneity are ruled out by cell cycle score regression according to Seurat manual.

### Clustering of Fast Growth and Slow Growth Subpopulations

We perform unsupervised clustering of single cell dataset using shared nearest neighbor (SNN) modularity optimization using FindClusters function of Seurat with resolution of one. These clusters are visualized using uniform manifold approximation and projection (UMAP) dimensionality reduction (McInnes et al., 2018).

We assign cell cycle score to each cell using the CellCycleScoring function of Seurat, which quantifies G2M and S phase scores of single cells based on the scoring strategy and the cell cycle marker genes suggested from previous studies (Kowalczyk et al., 2015; Tirosh et al., 2016). Then, each cell is classified as a cell in G2M, S, or G1 phase according to its cell cycle score.

The arrest signature score of each cell is quantified with AddModuleScore of Seurat along the cell cycle arrest related gene sets extracted from MSigDB (Subramanian et al., 2005; Liberzon et al., 2011, 2015). It is the gene set from Gene Ontology (GO) term, GO_REGULATION_OF_CELL_CYCLE_ARREST, that shows the most general coverage (Ashburner et al., 2000; The Gene Ontology Consortium, 2019). This gene set comprises 107 genes related with any process that modulates the rate, frequency, or extent of cell cycle arrest, the process in which the cell cycle is halted during one of the normal phases. Single cells are labeled as “arrested (Arr)” if their arrest signature scores are ranked higher than one-fifth of those of the whole single cells, otherwise labeled as “non-arrested (NArr).” If the ratio of Arr cells to NArr cells in a cluster is over 0.8 or less 0.2, then the cluster is labeled as “Arr” or “NArr,” respectively. A cluster with the average APC expression level smaller than the first tertile is labeled as “low APC,” otherwise it is labeled as “high APC.” Then a cluster with both “low APC” and “Arr” is defined as slow growth subpopulation (SG) and clusters with “low APC” and “NArr” are defined as fast growth subpopulations (FG).

In addition, we investigate on differential markers of the identified clusters using FindMarkers function in Seurat package (Butler et al., 2018; Stuart et al., 2019), by setting log fold change threshold to 0.25 (Supplementary Table S3). We also characterize the cell type change of shAPC single cell RNA-seq samples with differentially expressed genes (DEGs) between 1CT and 1CT-A to examine whether there is any new cell type appeared, but no distinctive patterns are observed. The DEGs between 1CT and 1CT-A are determined with two-fold change and 0.01 cutoff of value of *p* using two-tailed *t*-test.

### Characterization of Slow Growth Subpopulation

Apoptosis signature scores of SG cells are measured in the same way as the arrest signature score described in “Clustering of Fast Growth and Slow Growth Subpopulation” section, according to the cell apoptosis related gene set, GO_EXECUTION_PHASE_OF_APOPTOSIS (Ashburner et al., 2000; The Gene Ontology Consortium, 2019).

Stemness signature scores of SG cells are quantified using TCGAanalyze_Stemness function provided by TCGAbiolinks R toolkit, which generates mRNAsi stemness index described in the previous study (Malta et al., 2018). The stemness signature used here is PCBC_stemSig which is the default stemness signature obtained using the data from Progenitor Cell Biology Consortium (PCBC). We analyze whether there are subclusters within FG or SG along with the signature score, but the groups along with signatures are not clearly discriminated in the activity inference (Supplementary Figure S7).

### Protein Activity Inference Using VIPER

meta-Virtual Inference of Protein-activity by Enriched Regulon (metaVIPER) analysis is conducted to investigate the master regulators which control the fate determination of FG and SG (Alvarez et al., 2016). Since the single cell dataset lacks a tissue context, we use here metaVIPER which infers a regulatory network without tissue-specific regulatory information (Ding et al., 2018). First, the network of CRC cell is inferred using the patient expression dataset obtained from The Cancer Genome Atlas (TCGA) by the RTN package (Fletcher et al., 2013; Castro et al., 2016). Then, metaVIPER analysis is performed upon this CRC network with inputs composed of those genes of interest. The input gene lists used here are the list of DEGs between FG and SG (1.5-fold change, *p* < 0.01), and regulon lists generated by single-cell regulatory network inference and clustering (SCENIC).

### Reconstruction of Gene Regulatory Networks Using SCENIC

Single-cell regulatory network inference and clustering analysis is performed to generate the gene regulatory networks of SG and FG as described in the original paper using pySCENIC version 0.9.19 (Aibar et al., 2017). The corresponding auxiliary datasets used for SCENIC analysis are human cisTarget of 100 bp down, 500 bp up, and 10 kb up and down with the genome version of hg38, human TF binding motif provided by cisTargetDB of version 9, and the list of curated human TF comprising 1,390 genes. The resulting regulon lists are collected and used for the metaVIPER analysis to compute the activity difference between SG and FG.

### Selection of Targets

Target candidates generated from metaVIPER are filtered by *t*-test between SG and FG (*p* < 0.01). Then hierarchical clustering is performed with these genes to select a gene group downregulated in SG. The candidates are ranked along with the difference of average activity and expression level between SG and FG. The candidates of the top largest difference are selected for the next step analysis. At this point, since the expression level shows less clear discrimination between SG and FG, the threshold for expression difference is set to be one-third while that for activity difference is set to be one-sixth.

Next, the filtered target candidates are screened by how they are closely related to APC under the rationale that the target should cover the effect of APC in order to keep SG. Therefore, we investigate the shortest path length between APC and the target candidate by using the input list comprising APC, CTNNB1, WNT, candidate itself and its downstream target gene list produced by SCENIC in STRING DB version 11 (Szklarczyk et al., 2019). In addition, how much target genes a candidate shares with APC is quantified from the gene regulatory network inferred from CRC patients in TCGA database described in “Protein Activity Inference Using VIPER” section.

Then, interactions within target candidates are explored by MINDy to reconstruct a network composed of candidate TFs and their modulators (Wang et al., 2009). The most densely connected TF with others is considered as the most important master regulator.

Networks are visualized using Cytoscape version 3.7.1 (Shannon et al., 2003).

## Results

### Slowly Growing and Fast Growing Subpopulations Are Found From the scRNA-seq Dataset of APC-Deficient Normal Colon Epithelial Cells

**Figure 1:**
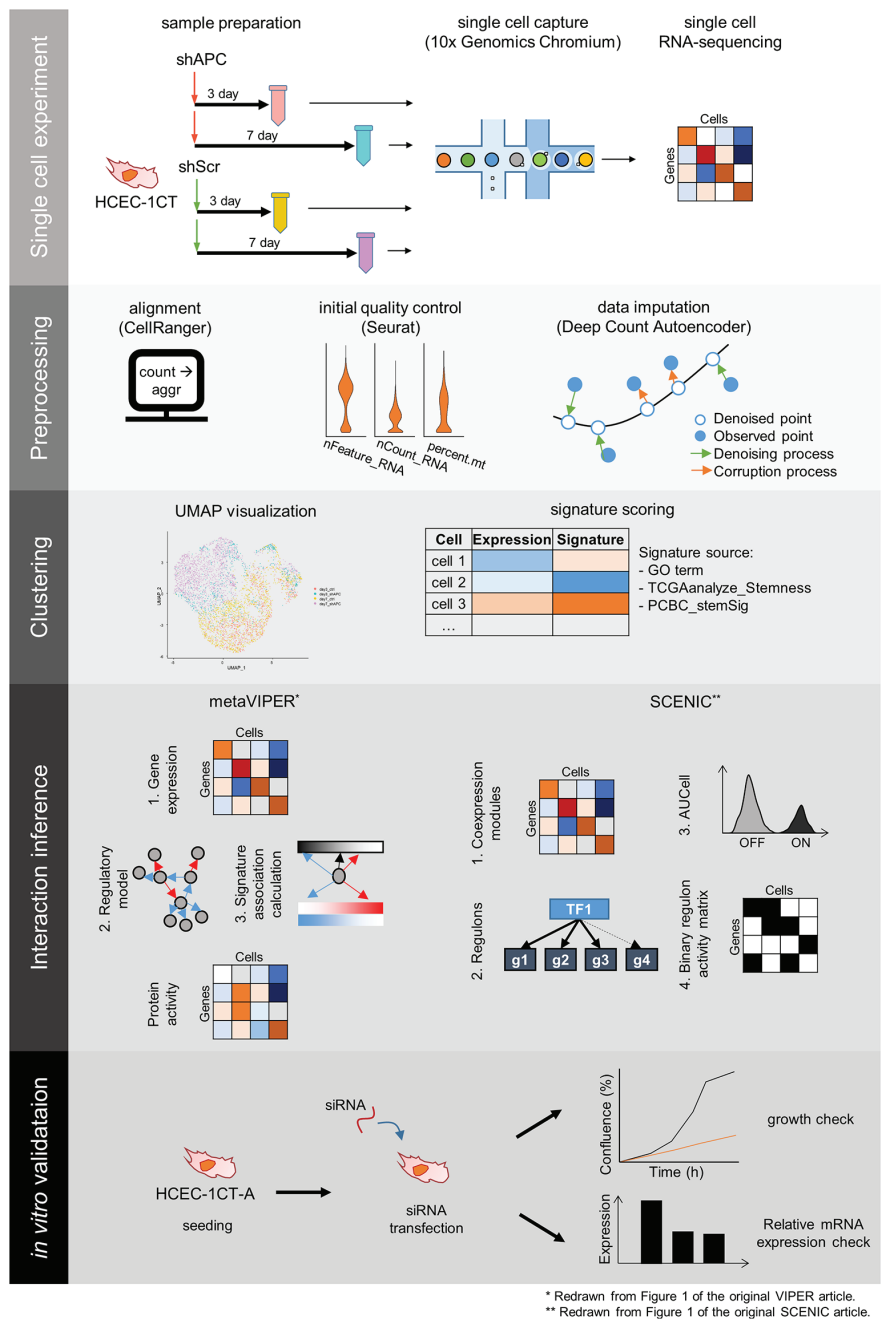
The scheme of single cell RNA-sequencing (scRNA-seq) experiment and analysis. scRNA-seq experiment and analysis comprise five steps: single cell experiment, preprocessing, clustering, interaction inference, and *in vitro* validation. Samples for scRNA-seq are prepared by transduction of shRNA targeting APC (shAPC) or scrambled shRNA (shScr) in HCEC-1CT cells, and scRNA-seq is performed using 10x chromium platform. Then we take preprocessing steps such as alignment, initial quality control, and data imputation. The single cell data points are clustered, and each cluster is scored according to gene signatures. Then, interactions within distinct clusters are inferred using Virtual Inference of Protein-activity by Enriched Regulon (VIPER) and single-cell regulatory network inference and clustering (SCENIC) to produce master regulators for the clusters. These master regulators are validated using siRNA transfection in 1CT-A cells.

**Figure 2:**
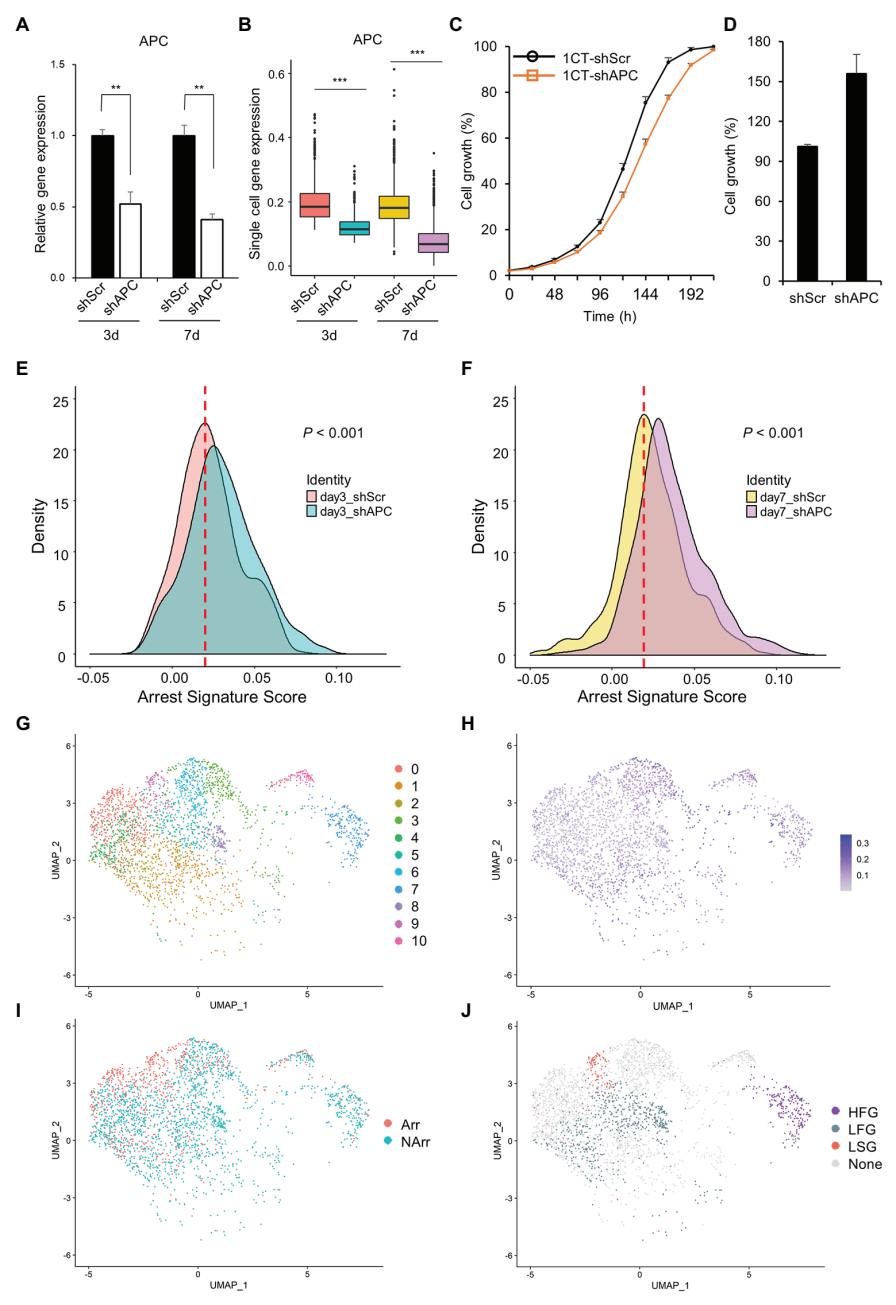
Clustering of slow growth and fast growth subpopulation in scRNA-seq dataset. **(A)** Relative adenomatous polyposis coli (APC) gene expression of single cell samples in quantitative reverse transcription PCR (qRT-PCR). **(B)** APC gene expression of single cell samples in scRNA-seq dataset. **(C)** Growth curve of HCEC-1CT with shScr and shAPC transduction during the short initial period of time (~7 days after transduction). The shAPC samples grow slightly slower than shScr samples. **(D)** Growth rate of HCEC-1CT with shScr and shAPC transduction (16 days after transduction). The shAPC samples grow faster than shScr samples. Distribution of arrest signature after APC knockdown **(E)** at day 3 scRNA-seq dataset and **(F)** at day 7 scRNA-seqe dataset. **(G)** Unsupervised clustering of scRNA-seq dataset. **(H)** APC gene expression level of scRNA-seq dataset. **(I)** Binarized arrest signature score of scRNA-seq dataset. **(J)** Cluster labels of scRNA-seq dataset. The criteria are designated to each cluster according to the combination of the APC level and arrest signature score: HFG for APC High and Fast Growth; LFG for APC Low and Fast Growth; LSG for APC Low and Slow Growth; and None for the remainders.

In order to investigate complex events occurring during the cancer initiation, we establish an early CRC development model, perform scRNA-seq, and analyze the scRNA-seq dataset (Figure 1). scRNA-seq is conducted at 3- and 7-days after transduction of shAPC or shScr on HCEC-1CT (1CT) cells. We examine the relative gene expression levels of APC and its downstream targets by performing qRT-PCR of remaining cells after single cell library preparation (Figure 2A and Supplementary Figure S2A), as well as by investigating the expression levels from scRNA-seq (Figure 2B and Supplementary Figure S2B). We confirm that the level of APC is dropped to at least 50% in shAPC samples compared to shScr samples in both bulk and single cell data.

Interestingly, we find that APC knockdown of 1CT decreases the cell growth mildly during a short initial period of time (about 7 days elapsed after shAPC transduction; Figure 2C) but eventually increases the cell growth at a later time (16 days elapsed after shAPC transduction; Figure 2D). This relationship between depletion of APC and the relatively slow cell growth is partially supported by a previous study reporting that APC loss drives the growth arrest or senescence program in the premalignant renal tumor (Cole et al., 2010). Since this trend is not observed in bulk qRT-PCR results (Figure 2C), we assume that it might be originated from rare and hard-to-observe events during CRC initiation.

To check out this assumption, we initially analyze changes in the arrest signature score between APC deficient cells and others, and find that the arrest signature score is increased in shAPC samples compared to that of shScr samples (Figures 2E,F and Supplementary Figure S3). This shift of the arrest signature score appears in both day3 and day7 samples, and becomes clearer in day 7 samples.

In order to figure out the source of driving this increased arrest signature in APC downregulated cells, we investigate the characteristics of clusters in shAPC single cell samples by assuming that there might be a subpopulation responsible for this phenomenon. Eleven clusters are identified and labeled according to four criteria (HFG for APC High and Fast Growth; LFG for APC Low and Fast Growth; LSG for APC Low and Slow Growth; and None for the remainders) based on arrest signature and APC level (Figures 2G–J and Supplementary Table S2). There are one cluster of HFG (Cluster 7), three clusters of LFG (Clusters 2, 5, and 8), and one cluster of LSG (Cluster 9). It is remarkable that the population of LSG is about one-sixth of LFG population, which is the reason why bulk analysis could not capture the characteristics originated from LSG (Supplementary Table S4 and Supplementary Figure S2A). Since our interest lies on the cells affected by APC downregulation, we exclude the HFG cluster in downstream analysis and take only LFG and LSG for further analysis. The labels of LFG and LSG are shortened hereafter as FG (Fast Growth) and SG (Slow Growth), respectively.

### SG and FG Have Different Phenotypical Characteristics and Gene Regulatory Networks

**Figure 3:**
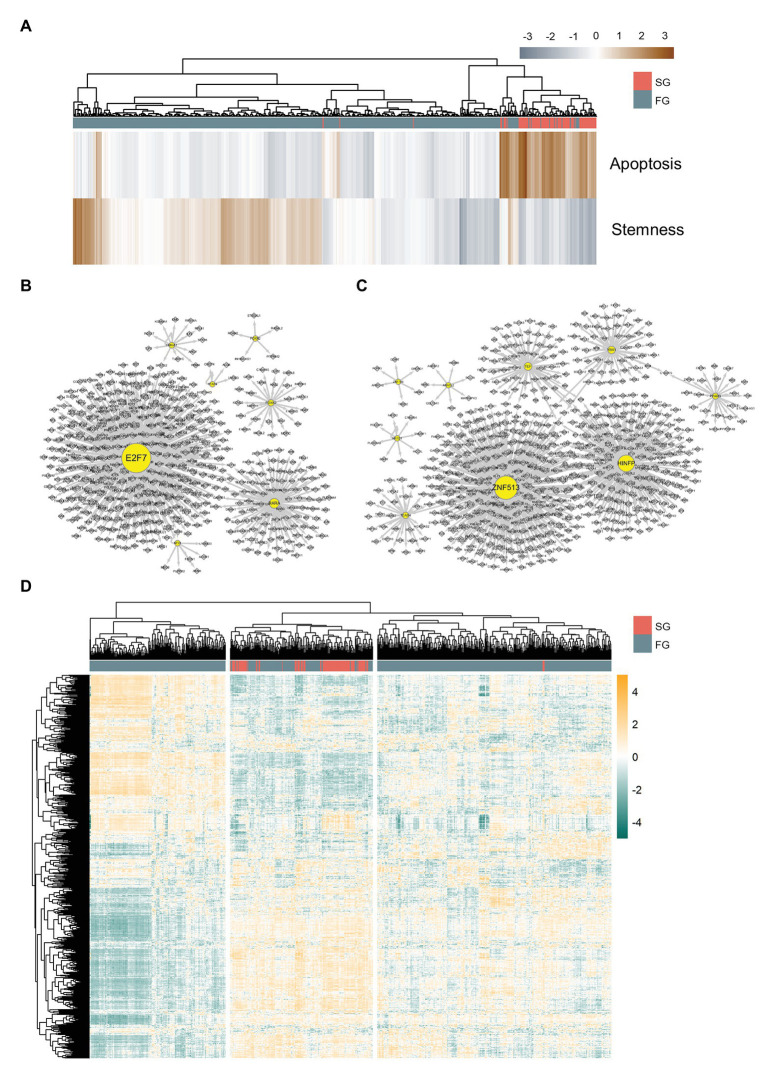
Differently organized gene regulatory networks of slow growth subpopulation (SG) and fast growth subpopulation (FG). **(A)** Apoptosis and stemness signature scores of SG and FG. Gene regulatory networks of **(B)** FG and **(C)** SG. **(D)** Master regulator analysis heatmap for SG and FG. SG and FG have different patterns of master regulator expressions.

We further examine the characteristics of SG and FG to see whether they actually differ in phenotypical biological processes such as apoptosis and stemness besides the arrest signature. As a result, we find that SG has a higher apoptosis signature and a lower stemness signature than FG (Figure 3A and Supplementary Figure S4), implying that SG has a fate to go through apoptosis without developing further malignancy.

Since SG is assumed to eventually diminish while FG is to progress into advanced cancer, we perform master regulator analysis to identify transcription factors that can drive FG to SG such that the majority of APC deficient cells undergo apoptosis. We perform metaVIPER (Alvarez et al., 2016; Ding et al., 2018) analysis with 848 DEGs between 1CT and its wild type APC depleted version, HCEC-1CT-A (1CT-A), and, as a result, we find 412 master regulators present in either SG or FG.

For further master regulator analysis, we examine the gene regulatory networks of FG and SG using SCENIC (Aibar et al., 2017) in order to determine whether the two subpopulations have differently organized gene regulation structures. Here, we define SG regulons and FG regulons as those genes shared by both regulons inferred from SCENIC and the master regulators found from DEG metaVIPER. It turns out that FG regulons comprise seven transcription factors such as E2F7, FOXN2, TFAP4, FOXK2, NFIX, RARA, and HMGA1 (Figure 3B), whereas SG regulons comprise nine transcription factors such as ARNTL2, YBX1, ZNF513, HINFP, PPARG, TEF, TEAD4, ZNF766, and NR1D1 (Figure 3C).

**Figure 4:**
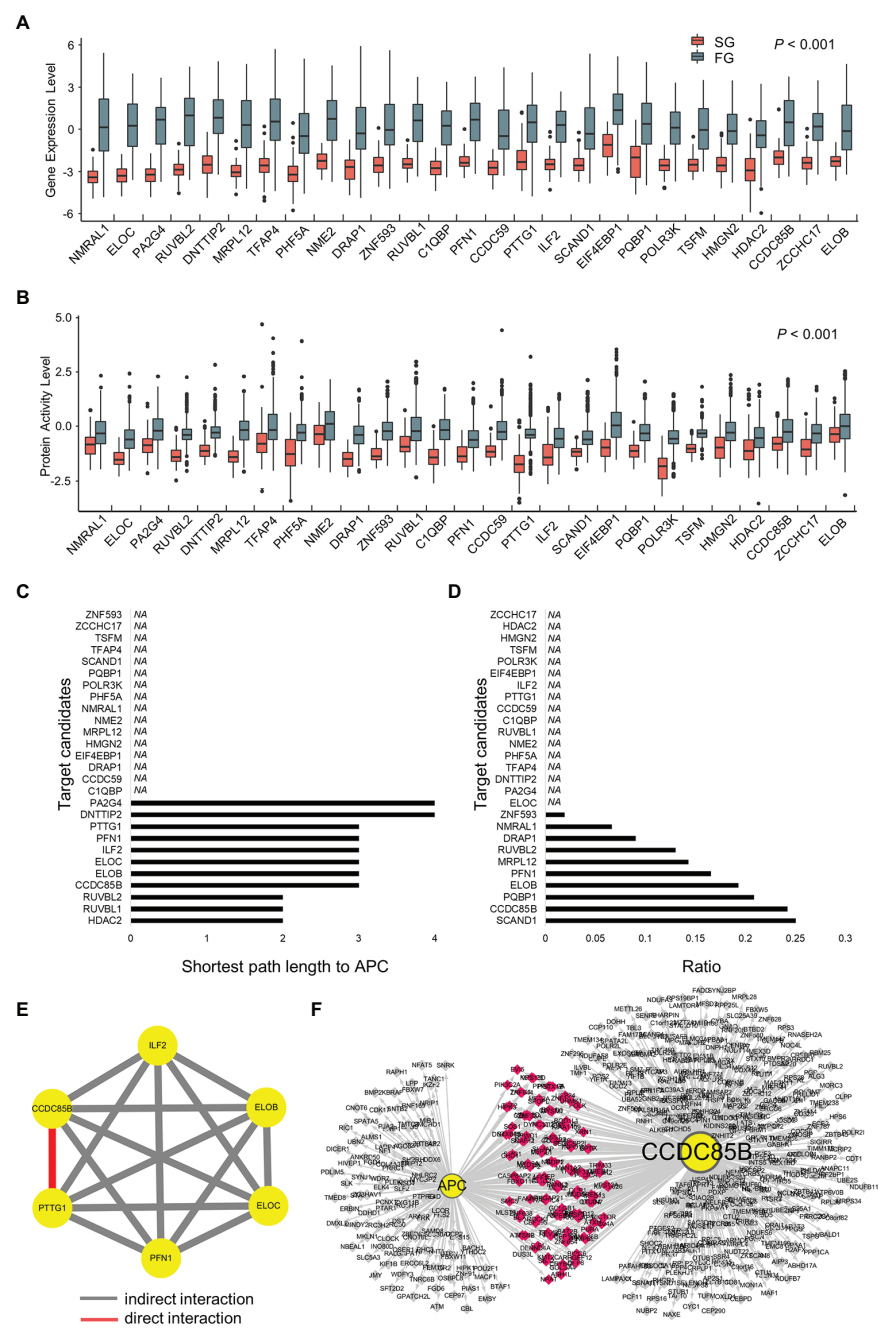
Prioritization of target candidates according to interactions with APC and the interactions among the candidates. **(A)** Gene expression levels and **(B)** protein activity levels of target candidates. **(C)** The shortest path length from each target candidate to APC. **(D)** Ratio of shared regulons between target candidates and APC. **(E)** Modulatory interactions within six target candidates. **(F)** Shared target genes of CCDC85B and APC.

We expand the list of regulons up to 1,411 genes by merging SG regulons, FG regulons, and their target genes. Then, we perform metaVIPER again with this list of regulons to find out the master regulators that can drive FG into SG (Figure 3D). Genes downregulated in SG with statistical significance (two-tailed *t*-test, *p* < 0.05) are taken and they are filtered again according to the level of difference in their expressions and activities across SG and FG (Figures 4A,B).

### CCDC85B and PTTG1 Are the Most Important Master Regulators Responsible for the Difference Between SG and FG

In order to narrow down the final targets, we examine the interactions between APC and target candidates or within target candidates (Figures 4A,B). First, the shortest path lengths between candidates and APC are investigated using STRING DB (Szklarczyk et al., 2019), resulting in three groups of genes which have any connection to APC: HDAC2, RUVBL1, and RUVBL2 for a length of two; CCDC85B, ELOB, ELOC, ILF2, PFN1, and PTTG1 for a length of three; DNTTIP2 and PA2G4 for a length of four (Figure 4C). In addition to the shortest path lengths, we investigate the number of genes which candidates share with APC, and CCDC85B is found to be one of the most densely APC regulon sharing genes (Figure 4D).

Since HDAC2 is known to have many redundant functions, it is classified as a less attractive marker for early cancer development (Jurkin et al., 2011). Considering that 1CT cell line has hTERT manipulation, genes related with telomerase such as RUVBL1 and RUVBL2 might be screened as 1CT context specific targets. Therefore, the genes with the length of three are considered as more promising targets instead of those with the length of two, and their interactions *via* modulators are probed using Modulator Inference by Network Dynamics (MINDy; Wang et al., 2009; Figure 4E). As a result, we find that only CCDC85B and PTTG1 have a direct cross-modulation relationship among six candidate genes. Assuming that tightly bound master regulators are more likely to control the biological process that is distinct in each of SG and FG, we conclude that CCDC85B and PTTG1 can be the final target candidates.

Since CCDC85B and APC share 124 genes (Figure 4F) and their shared genes are participants of essential biological processes such as regulation of macromolecule biosynthetic process and regulation of RNA metabolic (Supplementary Table S5), we select CCDC85B as a primary target candidate.

To validate that CCDC85B and PTTG1 are relevant with the characteristics of SG, the correlation between their expressions or activities and the apoptosis or arrest signature are further investigated (Supplementary Figures S5, S6). Both activity and expression of CCDC85B have a negative correlation with arrest and apoptosis signature scores, implying that its downregulation might slow down the cell cycle. The relationship of the expression or activity of PTTG1 and the score of apoptosis or arrest is similar to that of CCDC85B.

### *In vitro* Knockdown of CCDC85B Shows Significant Influences on Both Stemness and Cell Cycle as Predicted by Network Analysis

**Figure 5:**
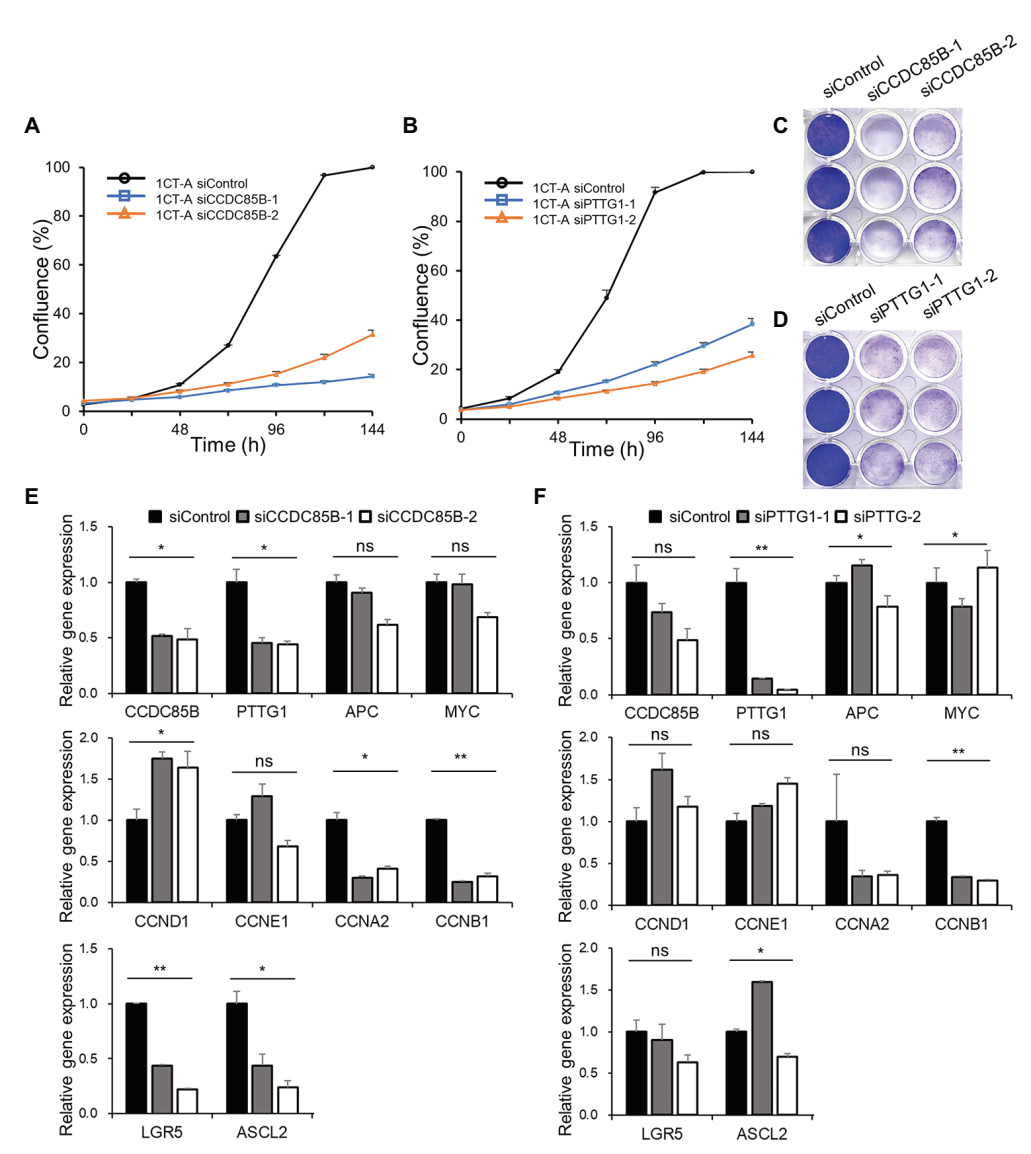
Growth curve and gene expression level after CCDC85B or PTTG1 interference in 1CT-A cells. Growth rate of 1CT-A cells after interfering with **(A,C)** CCDC85B or **(B,D)** PTTG1. Relative gene expression levels of 1CT-A cells after interfering with **(E)** CCDC85B or **(F)** PTTG1.

To validate whether the candidate targets can actually interrupt cancer progression, we perform *in vitro* knockdown of CCDC85B and PTTG1 using siRNA and examine the changes of the growth rate and transcriptomic levels. The cell growth rate of 1CT-A is dramatically decreased with the transfection of siRNA targeting CCDC85B (siCCDC85B; Figures 5A,C). The relative mRNA levels of APC and MYC are not affected by siCCDC85B transfection, whereas those of CCDC85B and PTTG1 are decreased a lot (Figure 5E). We investigate the changes in the level of various cyclins to figure out which cyclins CCDC85B has affected. Since siCCDC85B decreased the relative mRNA levels of Cyclin A2 and Cyclin B1, we can infer that CCDC85B might act on G2/M phase (Figure 5E). Interestingly, it coincides with the cell phase where the majority of SG stays in our single cell data (Supplementary Table S2).

Next, we examine the effects of PTTG1 interference using siRNA (siPTTG1) in 1CT-A on cell cycle and stemness since PTTG1 is considered to function with CCDC85B according to MINDy analysis. The cell cycle is arrested by siPTTG1 transfection, and the primarily affected cyclins are Cyclin A2 and Cyclin B1 as in the case with the siCCDC85B results (Figures 5B,D). siPTTG1 transfection reduces only PTTG1 significantly not CCDC85B, whereas siCCDC85B transfection reduces the level of both CCDC85B and PTTG1. We need to note that stemness markers for colon cells such as LGR5 and ASCL2 show a non-significant change when PTTG1 is perturbed unlike CCDC85B perturbation experimental results (Figure 5F).

## Discussion

In this study, we investigate master regulators the downregulation of which can lead to suppression of early CRC progression by analyzing scRNA-seq data. We establish the early CRC development model by interfering with APC using shRNA in normal colon epithelial cells, 1CT, and then we conduct scRNA-seq to capture small and heterogeneous changes that occur during the earliest events in CRC initiation. Since increment of arrest signature after APC downregulation is observed, we assume that there might be subpopulations responsible for this shift. We find out two subpopulations with different growth rates, and define one subpopulation with a relatively slow cell cycle as the slow growth subpopulation (SG) and the other with a relatively fast cell cycle as the fast growth subpopulation (FG). Through further analysis, we find that SG and FG differ in their organization of gene regulatory networks, as well as cell growth rates. Interestingly, SG has a low stemness signature and a high apoptosis signature, whereas FG has a high stemness signature and a low apoptosis signature. Although there is no direct experimental evidence presented in this study, it is highly likely that SG eventually goes through apoptosis instead of developing malignancy by acquiring stemness contrasting to the opposite fate of FG. Hence, we presume that transforming the FG into the SG might be a useful strategy of restraining early CRC development as it pursues diminishing the cell population of a malignant fate.

From the master regulator analysis, we identify CCDC85B and PTTG1 as the two most promising master regulators that can discriminate SG and FG and validate that both can lower cell growth rates by knockdown experiments using siRNA. In particular, knockdown of CCDC85B lowers the expression level of stemness markers such as ASCL2 and LGR5 in addition to the level of cyclins, whereas knockdown of PTTG1 lowers only the expression level of cyclins. Both CCDC85B and PTTG1 affect Cyclin A2 and Cyclin B1, which are known to act at G2/M phase. This might be a predictable result since PTTG1 is previously reported to act as a master regulator that controls the cell cycle at G2/M phase (Quereda and Malumbres, 2009; Liang et al., 2011). It is noteworthy that HDAC2 is one of the differential master regulators between SG and FG besides CCDC85B and PTTG1, since it implies that chromatin regulation plays a role in the discrimination of SG and FG. Considering that APC is known for its contribution in the chromosomal instability seen in many colon cancer cells, it seems natural for chromatin regulation to appear as one of the controlling mechanism of the earliest events of cancer development. A further study on this relationship between chromatin regulation and characteristics of SG and FG would add more value to the understanding on the earliest events in CRC initiation.

We infer that CCDC85B regulates stemness and cell cycle *via* β-catenin and PTTG1, respectively, based on literature survey and our own experiments. It is known that CCDC85B is overexpressed in the tumor sample of non-small cell lung cancer patients and that CCDC85B takes a crucial part in activation of β-catenin (Feng et al., 2019). Therefore, we can infer the decreased level of colon epithelial stemness markers after CCDC85B knockdown might be a result of the decreased active β-catenin induced by CCDC85B knockdown.

We show that CCDC85B knockdown decreases the relative mRNA expression levels of both CCDC85B and PTTG1, and that CCDC85B and PTTG1 has similar effects on the identical cell cyclins such as Cyclin A2 and Cyclin B1. Thus, we can infer that CCDC85B affects cell cyclins through PTTG1.

In summary, we suggest CCDC85B as a novel potential therapeutic target for restraining early CRC progression by lowering both the cell growth rate and stemness through the regulation of PTTG1 and β-catenin.

## Data Availability Statement

All data generated or analyzed during this study are included in this published article (and its Supplementary Material files). The sequencing data discussed in this publication are deposited in NCBI’s Gene Expression Omnibus (Edgar et al., 2002) and accessible through GEO Series accession number GSE152746 (https://www.ncbi.nlm.nih.gov/geo/query/acc.cgi?acc=GSE152746).

## Author Contributions

K-HC designed the project and supervised the research. SL, CYH, and JC performed single cell RNA-sequencing experiment. J-RG and CYH provided analytical and experimental guidance. CYJ provided experimental support. JC and K-HC wrote and revised the manuscript. All authors contributed to the article and approved the submitted version.

### Conflict of Interest

The authors declare that the research was conducted in the absence of any commercial or financial relationships that could be construed as a potential conflict of interest.
