# Supplementary material for: A Systems Biology Approach to Identifying a Master Regulator That Can Transform the Fast Growing Cellular State to a Slowly Growing One in Early Colorectal Cancer Development Model
Source: Front Genet. 2020 Oct 8;11:570546. doi: 10.3389/fgene.2020.570546 (PMC7579420; doi:10.3389/fgene.2020.570546)
Supplement: Supplementary file 1 [file Data_Sheet_1.pdf]

Supplementary Material

1 Supplementary Figures

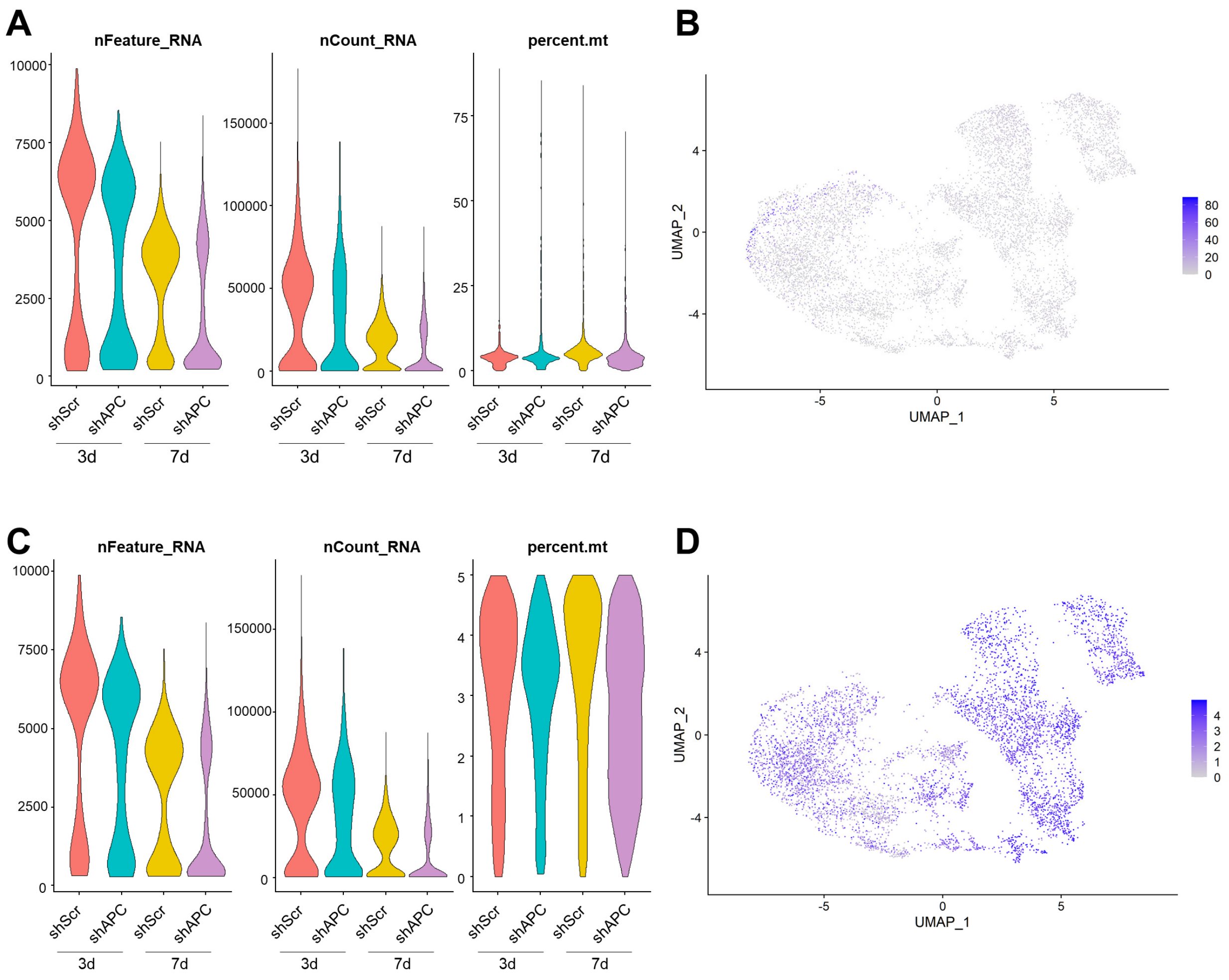

Supplementary Figure S1. Quality control metrics of single cell RNA-seq. Quality control (QC) metrics presented here are the number of unique genes detected in each cell, the total number of molecules detected within a cell, and the percentage of reads that map to the mitochondrial genome. Three QC metrics **(A)** before filtering cells with mitochondrial gene percentage over 5%, and **(C)** after filtering them. Distribution of mitochondrial gene percentage across the single cell data points **(B)** before filtering cells with mitochondrial gene percentage over 5%, and **(D)** after filtering them. There are few cells with exceptionally high mitochondrial gene percentage, removal of which does not affect the overall trend of QC.

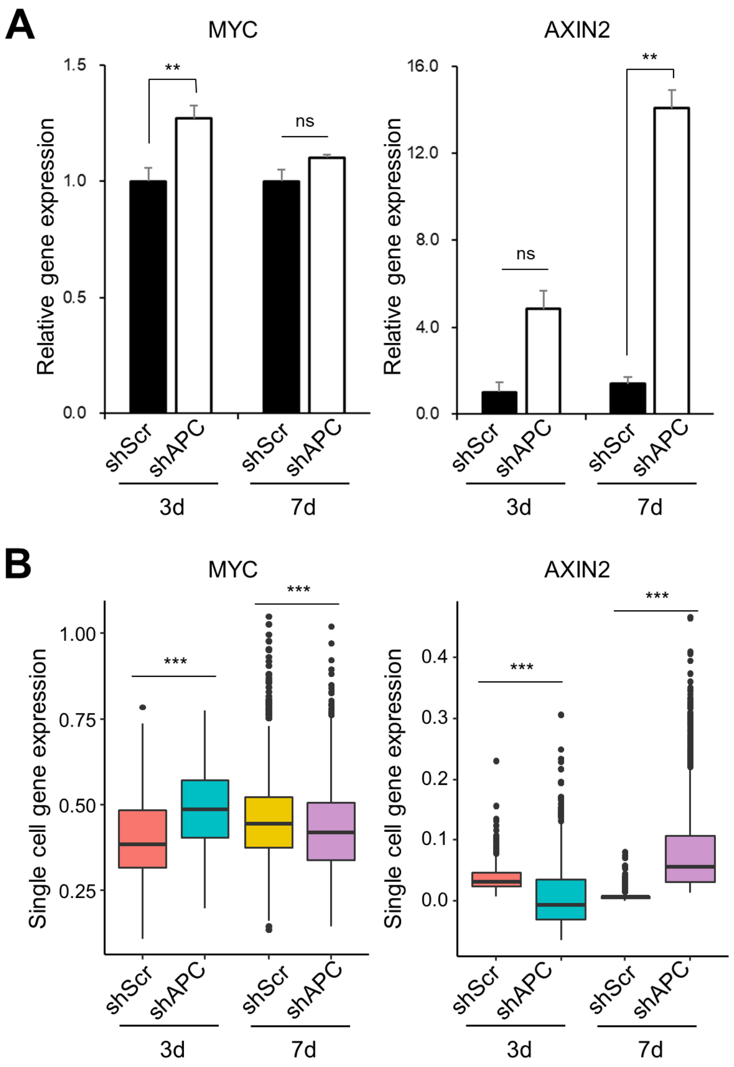

Supplementary Figure S2. Relative expression levels of APC downstream targets in bulk qRT-PCR and in single cell RNA-sequencing dataset. Relative expression in levels of MYC and AXIN2 in **(A)** bulk qRT-PCR of remaining cells after single cell library preparation, and in **(B)** single cell RNA-sequencing dataset. MYC level is relatively mildly increased, while AXIN2 level is dramatically elevated in shAPC samples in bulk data. However, both MYC and AXIN2 expression level trends are mixed in scRNA-seq data.

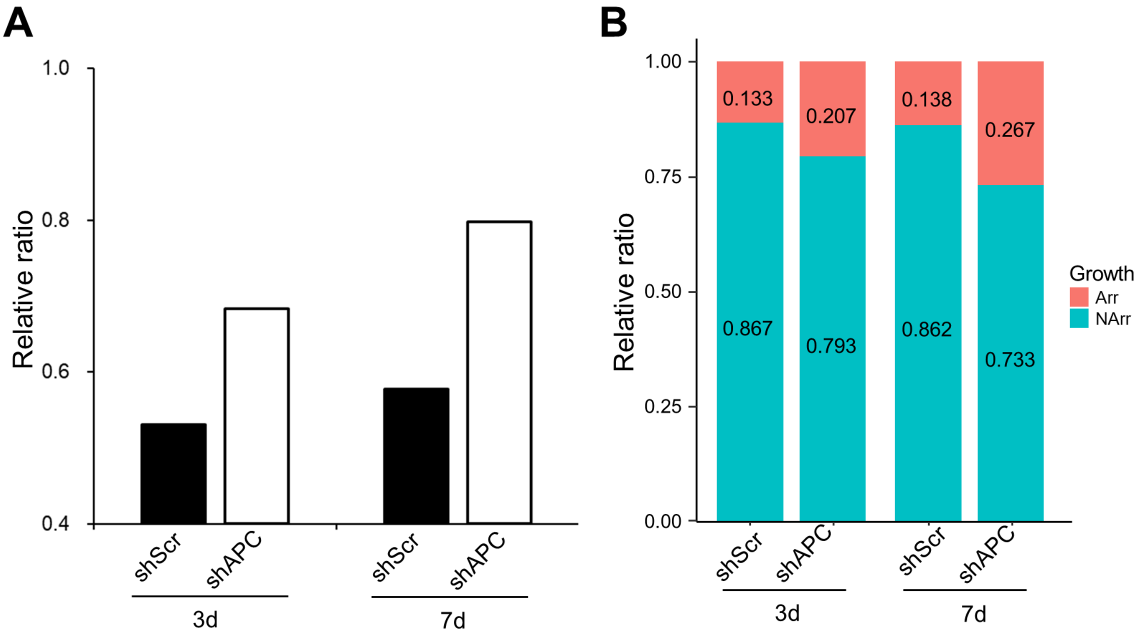

Supplementary Figure S3. Change in the arrest signature score between shScr and shAPC. (A) Relative ratio covered by area under curve after peak of arrest signature score distribution for each single cell RNA-sequencing samples. **(B)** Relative ratio of cells with higher arrest signature (>20%, Arr) versus the rest (NArr) for each single cell RNA-sequencing samples. Both graphs depict that the arrest signature scores of shAPC are increased compared to those of shScr.

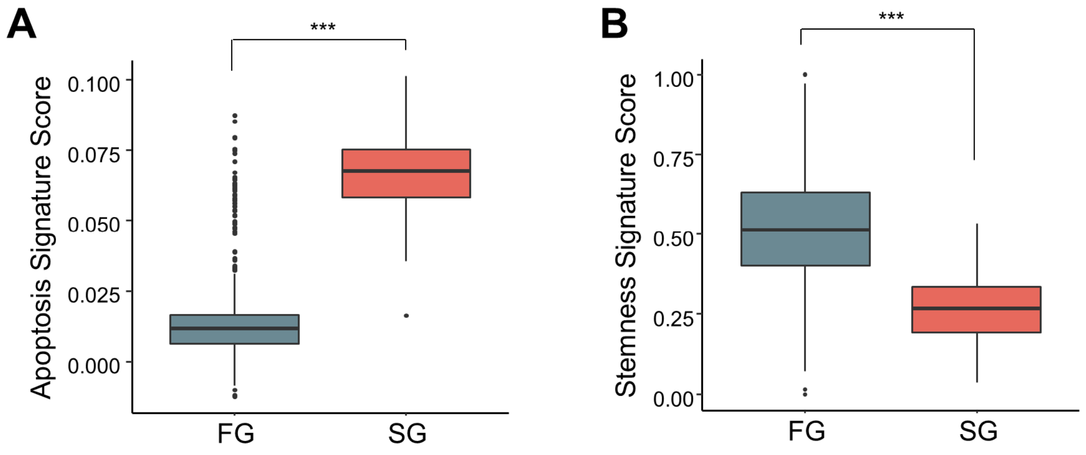

Supplementary Figure S4. Signature scores of FG and SG (A) apoptosis and **(B)** stemness signature of FG and SG. SG has relatively higher apoptosis signature score and lower stemness signature score than FG. It might imply that SG is destined to perish eventually while FG is inclined to develop further malignancy by acquiring stemness.

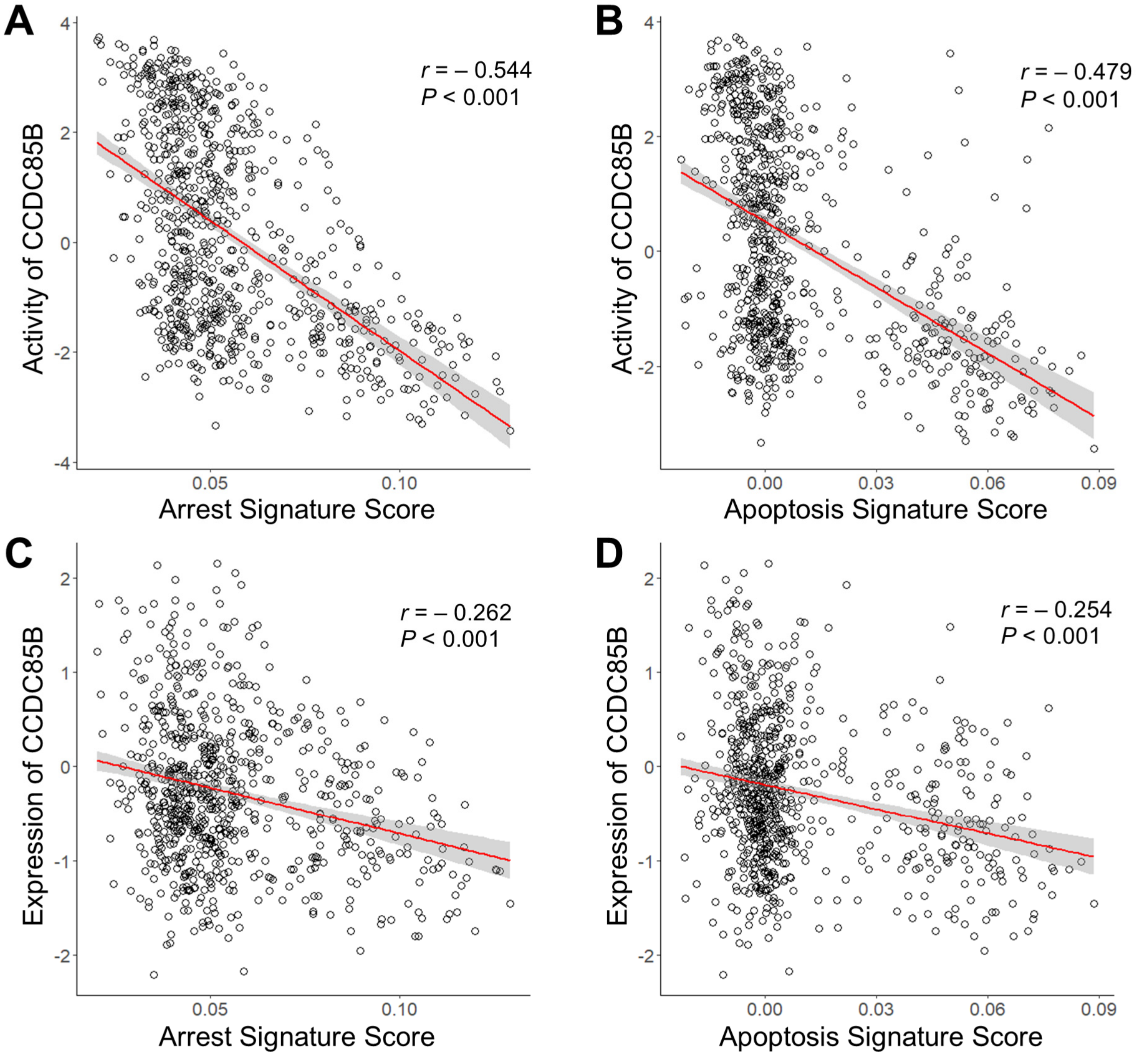

Supplementary Figure S5. Correlation between expression or activity of CCDC85B and score of apoptosis or arrest. Correlation between activity of CCDC85B and score of **(A)** arrest, or **(B)** apoptosis. Correlation between expression of CCDC85B and score of **(C)** arrest, or **(D)** apoptosis. Both activity and expression of CCDC85B have negative correlation with arrest and apoptosis signature scores, implying that its down-regulation might slow the cell cycle.

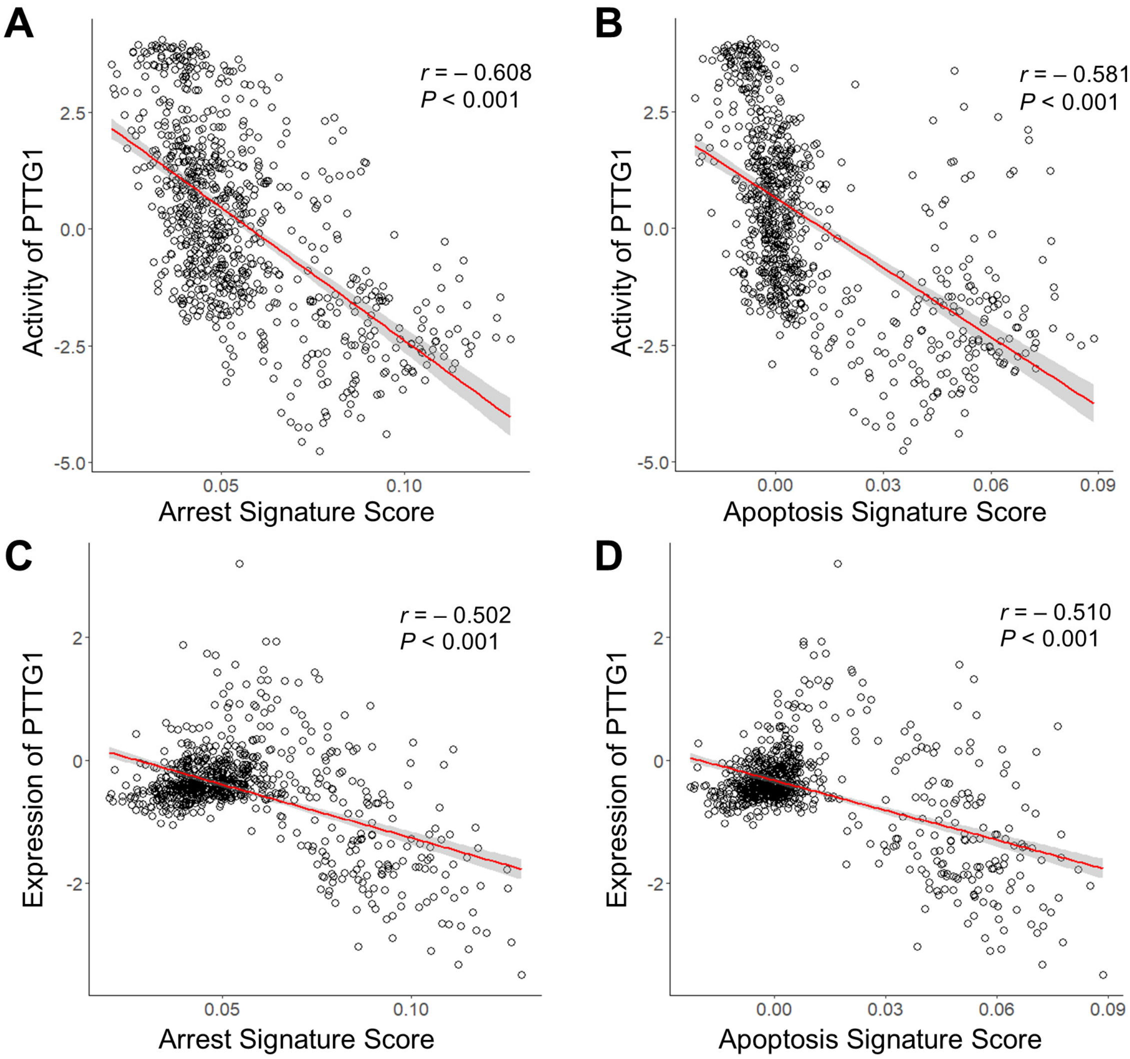

Supplementary Figure S6. Correlation between expression or activity of PTTG1 and score of apoptosis or arrest. Correlation between activity of PTTG1 and score of **(A)** arrest, or **(B)** apoptosis. Correlation between expression of PTTG1 and score of **(C)** arrest, or **(D)** apoptosis. The relationship of expression or activity of PTTG1 and score of apoptosis or arrest is similar as the case of CCDC85B, but display higher correlation. It is suggestive that PTTG1 has more direct relationship with cell cycle compared to CCDC85B.

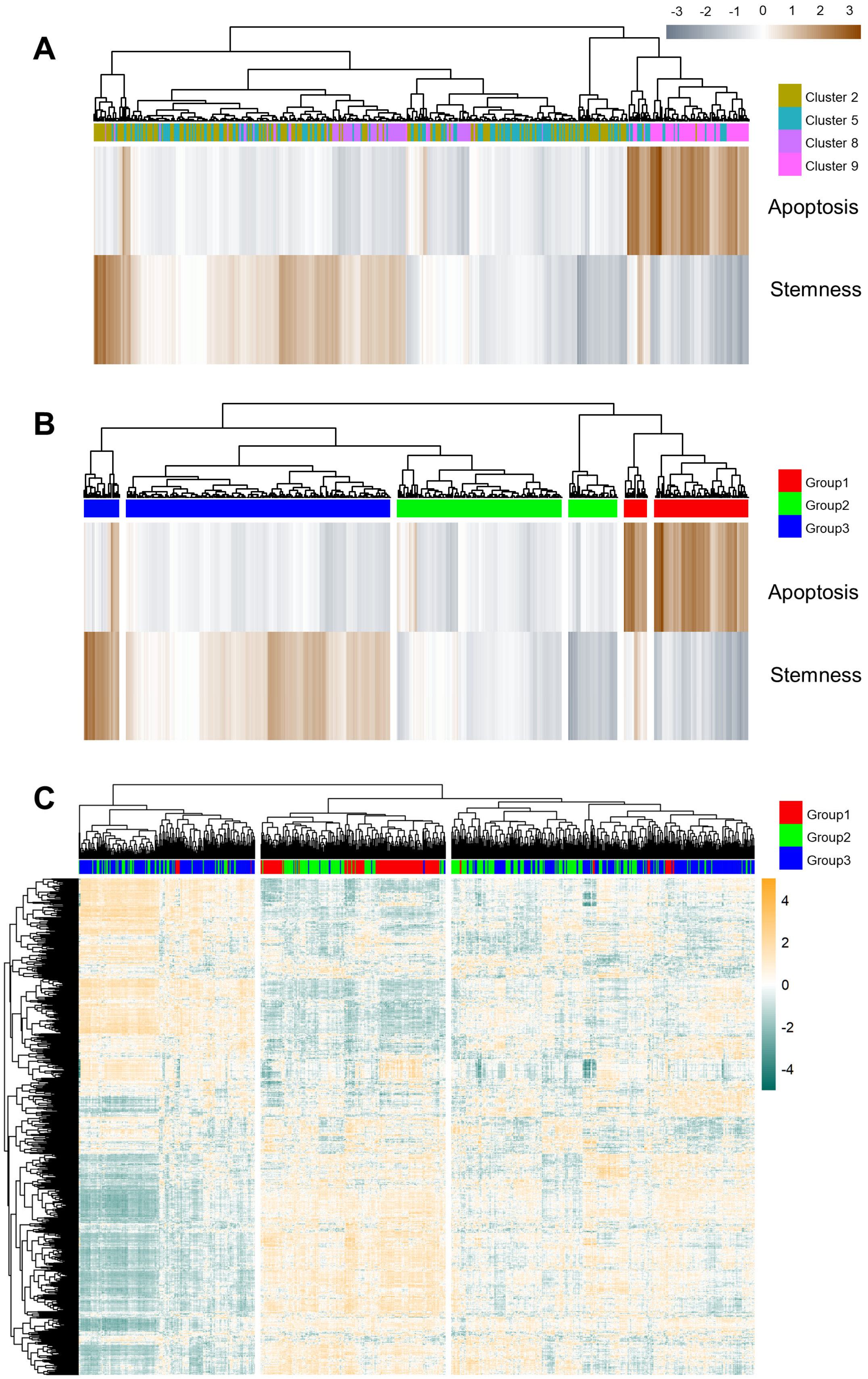
A
-3 -2 -1 0 1 2 3
Cluster 2
Cluster 5
Cluster 8
Cluster 9
Apoptosis
Stemness
B
Group1
Group2
Group3
Apoptosis
Stemness
C
Group1
Group2
Group3
4
2
0
-2
-4

Supplementary Figure S7. Analysis on subclusters of FG. (A) Original cluster mapping on signatures. **(B)** Groups along with signatures. (**C**) Mapping results of signature groups on activity heatmap. Group1 represents cells with high apoptosis and low stemness score; Group2 represents cells with low apoptosis and low stemness score; Group3 represents cells with low apoptosis and high stemness score. Original clusters which belong to FG (Cluster 2, 5, and 8) are mixed in both Group2 and Group3. Moreover, the groups along with signatures are not discriminated clearly in the activity inference. It seems that there are no subclusters in FG since the signature scoring and the inferred activities are not aligned.

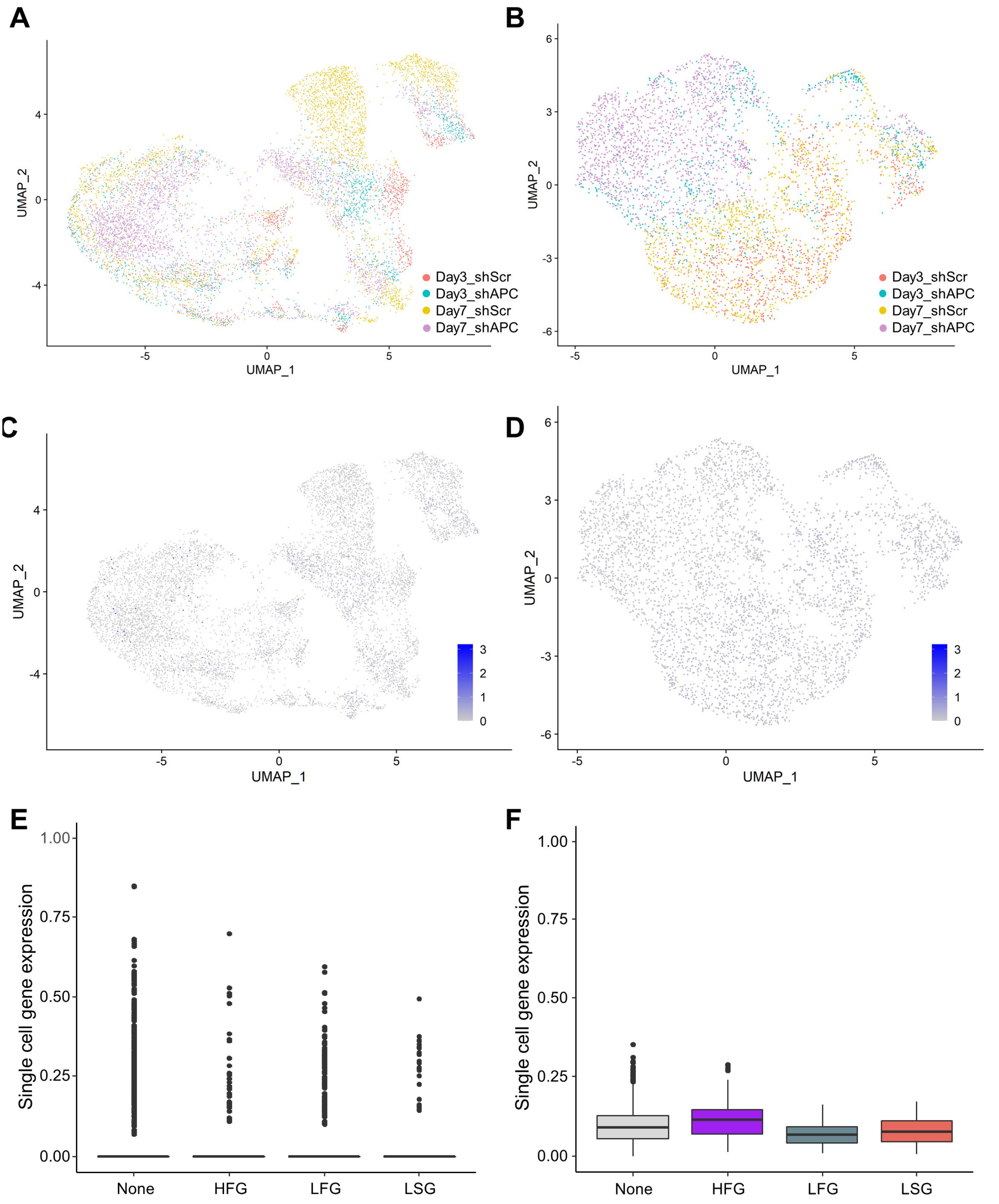

Supplementary Figure S8. Comparison of APC expression level before and after single cell data imputation with DCA. With single cell data before imputation, **(A)** the sample information, **(C)** APC expression level distribution in umap, and **(E)** box plot of APC expression level in four groups are

presented. After imputation, **(B)** the sample information, **(D)** APC expression level distribution in umap, and **(F)** box plot of APC expression level in four groups are changed.

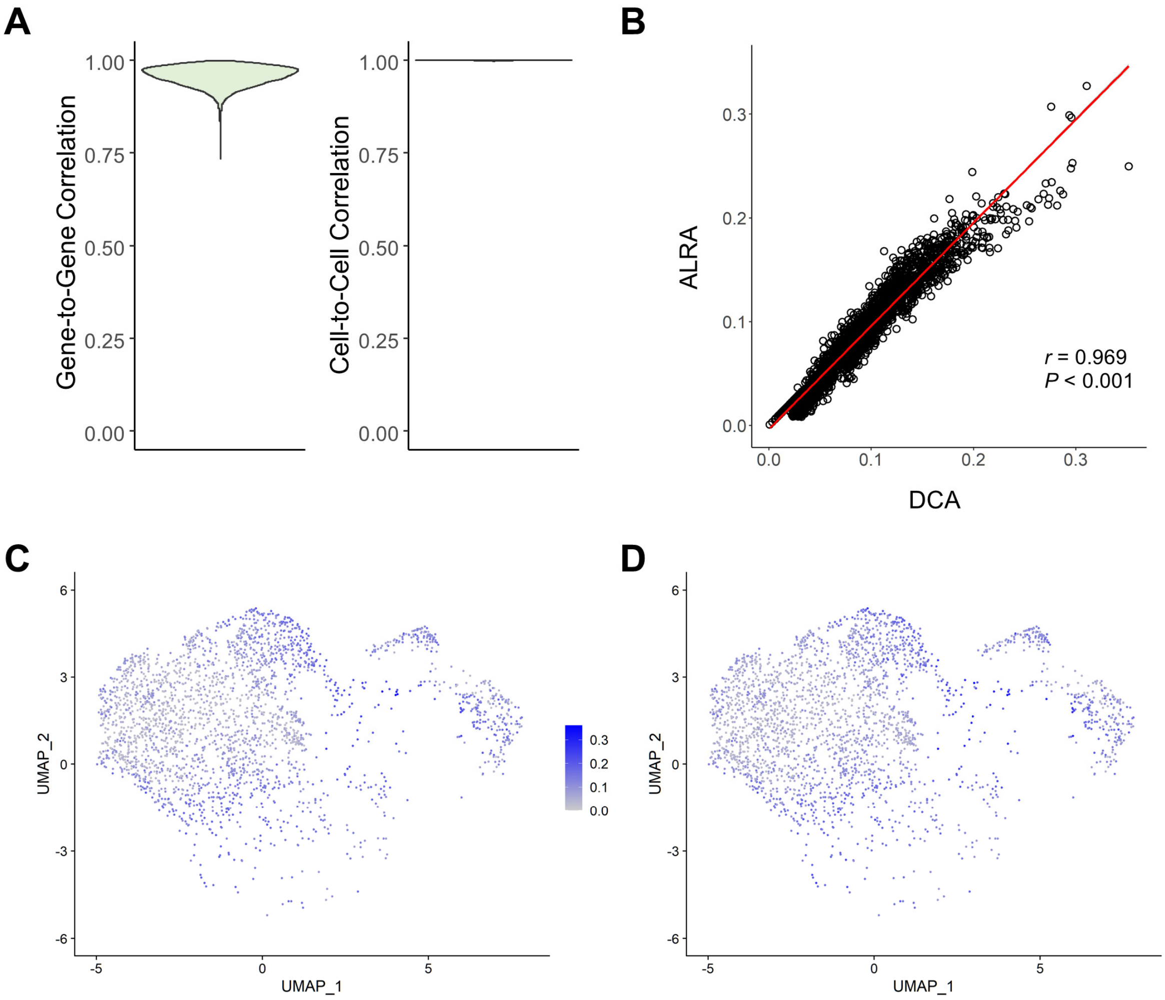

Supplementary Figure S9. Comparison of the results of different single cell data imputation approaches, ALRA and DCA. (A) Both gene-to-gene and cell-to-cell correlation between ALRA imputated data and DCA imputated data are high. **(B)** APC levels of ALRA imputated data and those of DCA imputated data are highly correlated, and APC level distribution patterns are similar **(C)** in ALRA imputated data and **(D)** in DCA imputated data.

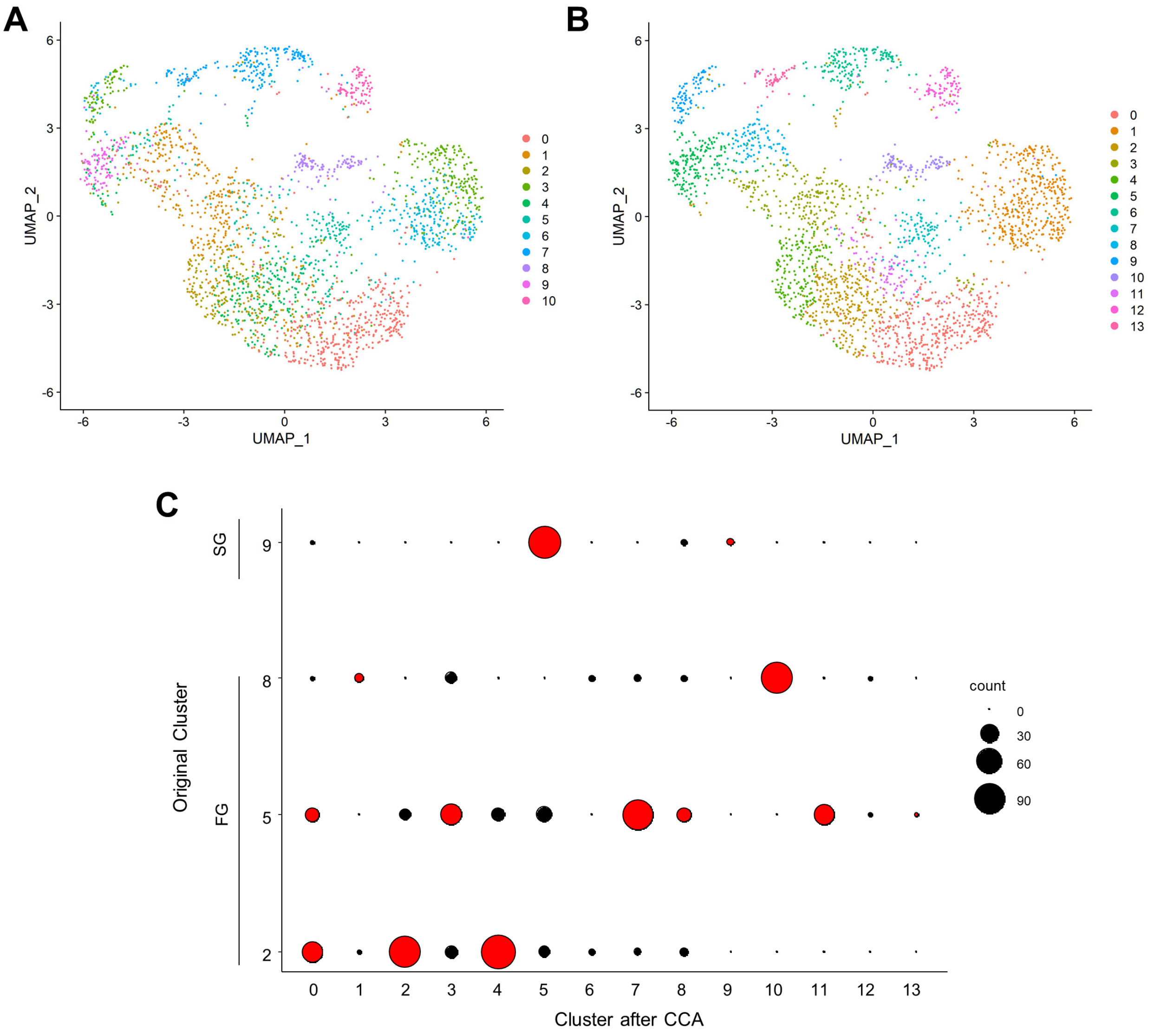

Supplementary Figure S10. Comparison of the results of different single cell data integration approaches, ComBat and CCA. Unsupervised clustering results with resolution of one with single cell data imputated with **(A)** ComBat, and **(B)** CCA. **(C)** Clusters of SG and FG are mapped to the clusters after running CCA (red: hypergeometric p value < 0.001). SG cluster and FG clusters are statistically discriminated even after using different integration process.

2 Supplementary Tables

Supplementary Table S1. Single-cell RNA sequencing data statistics. (Chemistry: v2 Chemistry (Chromium Single Cell 3'), pipeline: Cell Ranger v3.0.0, and reference: GRCh38).

Sample	Day3_shScr	Day3_shAPC	Day7_shScr	Day7_shAPC
Estimated Number of Cells from Cell Ranger	1,471	1,047	3,652	3,035
Mean Reads per Cell	139,538	199,228	57,356	76,108
Median Genes per Cell	4,076	5,900	909	3,592
Total Raw Reads	205,261,053	208,592,607	209,464,762	230,990,109
Mapping Rate to the GRCh38 Transcriptome (%)	68.6	66.1	74.5	71.2
Sequencing Saturation (%)	55.0	59.3	49.3	50.9
Final No. of Cells Analyzed post-processing	807	677	1,309	1,964
Total Genes post-processing	15,506	15,506	15,506	15,506
Mean Genes per cell post-processing	3167.672	3413.707	3302.426	3305.489
Mean UMIs per cell post-processing	15427.76	15427.76	15497.11	15489.87

Supplementary Table S2. Characteristics of clusters in scRNA-seq. Eleven clusters produced by unsupervised clustering are labeled with four criteria according to cell cycle phase, growth state, and APC level of each cluster's majority cell population. HFG for APC high and fast growth; LFG for APC low and fast growth; LSG for APC low and slow growth; None for everything else.

Cluster	Cell phase			Growth		APC level	Criteria
	G1	G2M	S	Arr	NArr		
0	384	21	6	209	202	0.059	None
1	233	102	42	79	298	0.122	None
2	253	19	19	28	263	0.081	LFG
3	211	66	9	68	218	0.141	None
4	205	22	35	58	204	0.060	None
5	201	42	8	40	211	0.063	LFG
6	215	23	4	60	182	0.090	None
7	117	51	36	17	187	0.112	HFG
8	113	3	4	4	116	0.062	LFG
**9**	**1**	**89**	**17**	**106**	**1**	0.080	**LSG**
10	79	3	8	33	57	0.109	None

Supplementary Table S3. Differential markers for each cluster in scRNA-seq. Differential marker genes for the eleven identified clusters were detected. (logfc threshold: 0.25, Wilcoxon Rank Sum p value < 0.005) The ten most statistically significant markers for each cluster are taken here.

**Cluster**	**Gene**	**avg_logFC**	**p_val_adj**
0	LAMA4	0.276798	2.58E-131
0	TP53INP1	0.268599	2.34E-129
0	MT-ND1	0.287028	9.24E-128
0	MTRNR2L8	0.274083	1.88E-127
0	MT-ND4	0.303578	1.80E-120
0	STOM	0.355918	1.06E-116
0	SLC7A11	0.398774	9.63E-115
0	GABARAPL1	0.307075	7.21E-111
0	SNHG25	0.263453	3.31E-92
0	SNHG19	0.264591	1.29E-60
1	BOLA3	0.320833	4.52E-102
1	PPP1R14B	0.361448	6.37E-101
1	CAV2	0.322066	7.05E-97
1	RPL22L1	0.495301	9.53E-97

**Cluster**	**Gene**	**avg_logFC**	**p_val_adj**
6	RHOQ	0.267369	4.71E-81
6	SNHG18	0.256007	9.64E-81
6	SOX4	0.27998	5.05E-79
6	ARL4C	0.254696	5.49E-79
6	PSAT1	0.300172	8.51E-78
6	MEG3	0.328678	1.85E-66
6	SERPINH1	0.262875	4.76E-62
6	INHBA	0.301912	7.83E-48
7	UBL5	0.29623	7.85E-55
7	LAPTM4A	0.293765	1.35E-50
7	NCL	0.356122	5.14E-50
7	HSPD1	0.358793	9.70E-47
7	CALD1	0.300563	9.29E-45
7	SRSF7	0.279495	1.41E-44

1	SET	0.304789	4.37E-90	7	HSPA8	0.258485	4.06E-43
1	PTMA	0.326851	5.74E-79	7	HINT1	0.303036	4.87E-43
1	COL8A1	0.414476	8.70E-79	7	CDA	0.250103	6.90E-40
1	LSM5	0.251083	1.16E-68	7	MICA	0.25808	2.72E-33
1	CAP1	0.253108	4.58E-55	8	RPSA	0.270326	4.97E-43
1	FBN2	0.332803	2.34E-41	8	EIF3D	0.32709	2.19E-42
2	ABLIM3	0.319336	2.85E-110	8	RPS7	0.262734	1.91E-41
2	CXCL5	0.391902	1.69E-94	8	RPS4Y1	0.324672	2.09E-41
2	CXCL6	0.430199	2.35E-92	8	EEF1D	0.270691	9.41E-37
2	CSF3	0.285308	3.74E-73	8	TMSB4X	0.336107	1.15E-36
2	MAP2	0.345469	8.97E-72	8	ATP5MC2	0.275294	2.09E-35
2	HMGA2	0.342082	1.35E-56	8	PABPC1	0.273345	2.85E-29
3	FBN1	0.288627	3.29E-90	8	NPM1	0.399734	1.74E-28
3	COL5A2	0.28252	1.22E-89	8	EEF2	0.257825	9.25E-18
3	CCDC85B	0.264529	3.17E-86	9	CAMK2N1	0.298734	1.18E-34
3	SFRP1	0.384911	2.91E-82	9	SMTN	0.273041	3.80E-33
3	ELL2	0.28107	2.05E-53	9	ACTN4	0.256053	1.93E-31
3	KRTAP2-3	0.300618	2.83E-52	9	ANP32B	0.262526	9.93E-31
3	THY1	0.253666	4.08E-49	9	HNRNPA3	0.285988	1.17E-30
3	CD59	0.262299	1.75E-47	9	LSM3	0.262333	3.22E-30
3	CAV1	0.279052	1.54E-44	9	SPATS2L	0.269876	5.79E-29
3	SH3BGRL3	0.253948	8.80E-31	9	TAGLN2	0.301299	3.80E-28
4	PGAM1	0.276155	1.15E-67	9	SURF4	0.276957	5.49E-25
4	DCBLD2	0.42125	1.43E-64	9	ACTB	0.250045	2.33E-17
4	CKAP4	0.292117	2.74E-64	10	G0S2	0.421849	8.50E-25
4	CALU	0.271154	7.15E-58	10	TPM1	0.476125	4.34E-23
4	SMURF2	0.29846	2.51E-54	10	IFIT3	0.341605	1.76E-22
4	KRTAP1-5	0.409582	8.83E-54	10	UAP1	0.253511	1.84E-22
4	CRIM1	0.302253	1.32E-53	10	ERRFI1	0.405864	1.93E-22
4	OGFRL1	0.251152	1.73E-50	10	MRPL57	0.250089	1.70E-19
4	AHNAK	0.255319	6.28E-40	10	VCAM1	0.26849	1.21E-18
4	DAB2	0.282765	2.41E-33	10	SLC7A5	0.263164	7.20E-18
6	TTC3	0.311125	6.92E-88	10	CDC42EP3	0.251517	1.03E-14
6	MMP14	0.323888	5.19E-82	10	CLIC4	0.263064	8.80E-14

Supplementary Table S4. Bulk RNA-seq dataset related to APC and its target genes and cell cycle related genes. Relative levels of APC and CCND1 are low in APC deficient cells, HCEC-1CT-A, and those of MYC, AXIN2 and other genes were high.

Gene symbol	Fold Change	p-value
APC	0.681	0.002
MYC	2.482	0.006
AXIN2	2.242	0.242
CCND1	0.262	0.001
CCNE1	0.731	0.001
CCNA2	2.380	0.000
CCNB1	1.715	0.270
CDKN1A	0.435	0.001
CDKN1B	1.604	0.009

Supplementary Table S5. GO term analysis of target genes of CCDC85B. 512 target genes of CCDC85B are participants of essential biological processes such as cellular metabolic process and translational process. The shared 124 target genes between CCDC85B and APC are mostly found in metabolic process (FDR<0.001, fold enrichment>1.5).

GO biological process complete	Fold enrichment	FDR
cellular metabolic process (GO:0044237)	1.53	5.31E-15
cellular nitrogen compound metabolic process (GO:0034641)	1.95	8.28E-14
macromolecule metabolic process (GO:0043170)	1.58	9.92E-13
nitrogen compound metabolic process (GO:0006807)	1.52	1.72E-12
nucleobase-containing compound metabolic process (GO:0006139)	2.02	2.42E-12
heterocycle metabolic process (GO:0046483)	1.97	3.05E-12
nucleic acid metabolic process (GO:0090304)	2.15	4.31E-12
gene expression (GO:0010467)	2.19	4.66E-12
cellular macromolecule metabolic process (GO:0044260)	1.64	1.06E-11
cellular aromatic compound metabolic process (GO:0006725)	1.93	1.41E-11
RNA metabolic process (GO:0016070)	2.35	4.23E-11
organic cyclic compound metabolic process (GO:1901360)	1.8	1.86E-09
cellular protein metabolic process (GO:0044267)	1.71	1.91E-09
translation (GO:0006412)	3.88	7.50E-09
cellular nitrogen compound biosynthetic process (GO:0044271)	2.22	9.46E-09
cellular macromolecule biosynthetic process (GO:0034645)	2.17	1.32E-08
protein targeting (GO:0006605)	3.9	2.10E-08
peptide biosynthetic process (GO:0043043)	3.65	3.24E-08
RNA catabolic process (GO:0006401)	4.64	4.11E-08
macromolecule biosynthetic process (GO:0009059)	2.1	4.18E-08
RNA processing (GO:0006396)	2.61	4.58E-08
ncRNA processing (GO:0034470)	3.53	6.34E-08
peptide metabolic process (GO:0006518)	3.21	6.37E-08
